# Resampling-Detection-Network-Based Robust Image Watermarking against Scaling and Cutting

**DOI:** 10.3390/s23198195

**Published:** 2023-09-30

**Authors:** Hao-Lai Li, Xu-Qing Zhang, Zong-Hui Wang, Zhe-Ming Lu, Jia-Lin Cui

**Affiliations:** 1 EFORT Intelligent Equipment Co., Ltd., Shanghai 201600, China; lihaolai@efort.com.cn; 2 School of Aeronautics and Astronautics, Zhejiang University, Hangzhou 310027, China; zhang_xq@zju.edu.cn; 3 Center for Generic Aerospace Technology, Huanjiang Lab, Zhuji 311816, China; 4 School of Information Science and Engineering, NingboTech University, Ningbo 315100, China; cuijl_jx@163.com

**Keywords:** image watermarking, scaling robustness, cutting robustness, resampling detection network

## Abstract

Watermarking is an excellent solution to protect multimedia privacy but will be damaged by attacks such as noise adding, image filtering, compression, and especially scaling and cutting. In this paper, we propose a watermarking scheme to embed the watermark in the DWT-DCT composite transform coefficients, which is robust against normal image processing operations and geometric attacks. To make our scheme robust to scaling operations, a resampling detection network is trained to detect the scaling factor and then rescale the scaling-attacked image before watermark detection. To make our scheme robust to cutting operations, a template watermark is embedded in the Y channel to locate the cutting position. Experiments for various low- and high-resolution images reveal that our scheme has excellent performance in terms of imperceptibility and robustness.

## 1. Introduction

Nowadays, with the rapid development of the Internet, multimedia information, especially images, can be seen everywhere. Despite it bringing convenience and variety to people, the wide usage of multimedia also causes the problems of privacy disclosure, information manipulation, and copyright infringement. Watermarking, however, is an excellent solution which can embed extra information into images imperceptibly and extract that information when necessary. It is also a protection scheme that can be applied to various kinds of images, including photos, images, medical images [1], light-field images [2], 3D images [3], and so on.

A watermark can be embedded in the spatial domain, frequency domains, and other domains defined for specific purposes. Spatial-domain-based methods are the first proposed kind of watermarking method, but they always have weaker performance. As described in [4], statistical features, like the shape and mean of histograms, are robust under different types of attacks and can be used to embed watermarks. This method is mathematically invariant to scaling the size of the images, independent of the pixel position in the image plane, statistically resistant to cropping, and robust to interpolation errors during geometric transformations and common image processing operations. However, histogram-based watermarking suffers from its limitation of histogram equalization since this operation will greatly distort the histogram shape. In 2019, Abraham and Paul [5] proposed a spatial-domain-based method which embeds the watermark by gradually spreading the information over a region of pixels. This method is designed for high image quality and high robustness to attacks. However, this method is not robust to rotation attacks and translation attacks. Frequency-domain based methods are the most popular ways in recent years. Discrete wavelet transform (DWT), discrete Fourier transform (DFT), and discrete cosine transform (DCT) are the most widely and effectively used frequency domains, and many recently proposed methods have combined them together or improved them to obtain better performance. Kamili et al. [6] proposed a dual-watermarking framework that embedded robust watermarks in the DCT domain and obtained strong robustness against cropping and resizing attacks. It should be pointed out that this dual-watermarking framework is designed for content authentication and tamper localization for industrial images. The robust and fragile watermarks along with overhead bits related to the cover image for tamper localization are embedded in different planes of the cover image. The results obtained confirmed that the scheme can stand firm against different singular as well as hybrid attacks and acceptably trace the regions being tampered as well. To achieve high robustness, many schemes make use of dual transforms. In [7], a blind robust watermarking scheme has been proposed, which is a blend of DWT and DCT. The watermark is first scrambled using the Arnold transform and then embedded in spread spectrum patterns with the help of pseudorandom series. The midfrequency DCT coefficients of the LL subband obtained after using DWT have been used for embedding. However, the use of dual transformations results in high computational cost. In [8], Hu and Hsu applied quantization index modulation (QIM) to DWT-DCT coefficients in an adaptive manner, where controlling parameters are designed to minimize the bit error rates of extracted watermarks subject to a quality criterion. However, this scheme cannot withstand desynchronization attacks. Because of the rapid development of watermarking schemes, original frequency-domain-based methods are unable to solve complex problems, and thus many new domains are defined. Kang et al. [9] proposed a near-uniform log-polar mapping (ULPM) domain to embed and extract watermarks, which showed robustness to geometric distortions and general print/scan processes. It is robust against RSTC distortion, general print/scan processes, and JPEG compression with low-quality factors simultaneously. However, this scheme has a low capacity. Liu et al. [10] proposed a method based on a new transform called DTCWT to resist geometric attacks. This method is robust against geometric attacks, such as cropping, rotation, scaling, shearing, and projective mapping. However, this method has a relatively high computational complexity.

Cutting attacks can be seen everywhere in our daily life when someone needs to highlight something or hide something. This type of attack will destroy the synchronization of the watermark, thus causing the failure of the whole watermarking scheme. Many schemes have been proposed to resist cutting attacks. The first type of schemes for resisting cutting operations embeds watermarks by uniformly distributing the information into the whole image. Su et al. [11] spread the watermark into small blocks and used the QR decomposition to embed each bit. This method focuses on embedding color watermark images into color host images. However, the robustness to JPEGs with high compression ratios, cropping of 50%, scaling of 1/4, median filtering, and Gussian noise is not so good. The second type of scheme embeds multiple copies of the watermark and uses redundant judgment to obtain the final watermark. The main problem of this kind of method is that the cutting attack will make the watermarks inconsistent, and thus it is difficult to find the correct beginning position. Hsu and Tu [12] proposed a dual-watermark scheme where they embedded a fragile watermark and a robust watermark simultaneously. The fragile watermark aims to locate the cutting position, and the robust watermark focuses on the information that needs to be embedded. However, this method is only designed for enhancing the robustness against cropping attacks and does not pay much attention to the robustness against other attacks, including common image processing operations.

In addition to cutting attacks, scaling attacks are also a very common but harmful attack in the watermarking field. When people use images, they usually need to shrink these images to save space or enlarge them to see more details. However, these operations will destroy the synchronization of the watermark. Zheng et al. [13] solved the scaling problem by using image normalization to transform regions into compact sizes that are scaling invariant. This scheme shows good performance in terms of the robustness against rotation, scaling, JPEG compression, and noise pollution. However, the capacity of this algorithm is not high. Kang et al. [14] embedded an extra template in the DFT domain and obtained the scaling factor by detecting this template. However, the robustness of the informative watermark against median filtering and random bending needs to be improved. Geometric invariant domains, like ULPM [9], were also applied to host images to resist scaling attacks. Another way to detect the scaling factor is based on image features. Bas et al. [15] proposed a geometrically invariant watermarking scheme using feature points. They performed Delaunay tessellation on the set of feature points and embedded the watermark in these triangles. However, the robustness of this scheme depends on the capacity of the feature point detector to preserve feature points after geometrical transformation, especially in highly textured images. Wang et al. [16] proposed a blind watermarking algorithm for dual-color images using discrete Hartley transform (DHT). It mainly used the image’s geometric features, such as sides and angles, to correct the attacked image and embeded a color watermark into a color image with large embedding capacity and strong practicability. However, this algorithm is not robust to rotation of 90°. Wang et al. [17] proposed a robust periodic blind watermarking scheme based on sub-block mapping and block encryption to enhance robustness under combined attack. The watermarked images were periodically encoded to raise the fault tolerance rate, and sub-block mapping and block encryption were also incorporated to enhance the security of copyright information and the visual quality of watermarked images. However, this algorithm is not very good at resisting scaling attacks.

The above-mentioned methods, however, need to embed extra watermark information or their geometric invariant ability is strongly related to the watermarking schemes. In the field of manipulation detection, resampling factor detection methods that can obtain the scaling factor only by the traces of scaling operations are commonly used, and they are suitable for watermark detection tasks. In 2005, Popescu and Farid [18] proposed a method to detect the resampling factor by employing the expectation-maximization (EM) algorithm. However, the major weakness of this approach is that it is only applicable to uncompressed TIFF images and JPEG and GIF images with minimal compression. Because of the large computation complexity and low speed, Kirchner [19] improved that work by calculating the gradient of the p-map spectrum to replace the EM algorithm. However, this modified detector is still vulnerable to recently presented geometric distortion attacks against resampling detection. When scaling an image, the interpolation operation will leave a peak in the DFT magnitude spectrum, so Gallagher [20] computed the second derivative and employed DFT to detect scaling factors. However, the performance of the interpolation detection algorithm decreases as the order of the interpolator increases. And sometimes the DFT signal will fail to produce meaningful peaks for some cases. Energy features have also been used; e.g., Feng et al. [21] proposed a method to extract the energy feature and train a support vector machine (SVM) classifier. However, the detection performance degrades with decreasing JPEG quality factors.

The development of deep learning gives the scaling factor detection task more possibilities. Luo et al. [22] proposed a method to train a dual-stream network that combines the features of gray images and differences in spectrum. However, this method is unable to detect the presence of resampling and estimate resampling parameters in the existence of more complex operation chains. Bayar and Stamm [23] analyzed traditional ways and found that most of them firstly obtained residuals by performing a filter and then subtracting the original image. As a result, they proposed a new layer called the constrained layer, which sums to zero and has −1 in the center, and put it as the first layer to construct the neural network. This method can perform general-purpose image manipulation detection; however, it cannot estimate the manipulation parameters. Ding et al. [24] focused on the energy feature and generated a measurable energy map toward the estimation of resampling factors. This method is outstanding for estimating the resampling rate; however, the problem of parameter estimation via deep learning for other image manipulations is still a potential and inspiring topic.

In the past few years, many deep-learning-based watermarking schemes have been proposed. The reason for using the neural network in watermarking is it enhances the watermarking efficiency compared to other methods. In [25], a hybrid watermarking scheme based on DWT and SVD in addition to a deep belief neural (DBN) network was proposed. However, this method does not perform well for image processing attacks with severe parameters. In [26], the learning ability of a deep learning network was utilized to automatically learn and generalize the watermarking algorithms and train them in an unsupervised manner to reduce human intervention. The employment of the embedding and extractor networks ensures that the proposed scheme is imperceptible and protects the mark image satisfactorily against attacks. However, the robustness to geometric attacks is not so good, and the embedding capacity is high.

It should be pointed out that in the past few years, many watermarking schemes have also emerged for copyright protection of deep learning networks. Recently, Fkirin et al. [27] have provided a comprehensive survey on digital watermarking methods for protecting deep neural networks. Unlike the work that focuses on using deep neural networks to aid the digital image watermarking process, this is related to another field, which uses digital watermarking methods to protect the copyright of deep neural network models, e.g, protecting the weights in deep neural networks. This is not our research topic.

Based on the above analysis, it is a hard but promising research task to design a robust image watermarking scheme with high imperceptibility that enables resistance to cutting and scaling attacks. To address this problem, this paper presents a robust digital image watermarking scheme based on deep learning with resampling detection and periodic head searching. This scheme can not only resist random cutting and scaling attacks, but it can enable extraction of the watermark from an image that has been attacked by both of these two attacks simultaneously. In addition, our proposed scheme also has high imperceptibility, enough capacity, and security assurance. It should be pointed out that our scheme is also based on the DWT-DCT domain, but the usage is different from the original DWT-DCT-based schemes [7,8]. In our scheme, the watermark embedding process mainly includes two parts: information embedding and template embedding, which are performed in the Cb and Y channels respectively. The information embedding process includes a two-order/two-dimensional DWT, block partition, block DCT, and coefficient quantization. And the template watermark embedding process is almost the same as the information embedding process, except we replace the two-order DWT with a one-order DWT for the reason that the lower level DWT can reduce the number of head searching points so we can extract the watermark faster. The main contributions of our method are as follows:

(1) We propose a watermarking method which can resist not only normal attacks but mixture attacks, like scaling and random cutting. Our method resists scaling attacks by using a scaling detection neural network, which is trained to focus on the detailed traces of the scaled images. In order to obtain the cutting position, a template watermark is embedded in the Y component and the position is found by maximal MSE searching.
(2) It is the first time to apply the resampling factor detection neural network to the watermark extraction area. Although deep learning has already been applied to enhance the robustness of digital image watermarking schemes, the deep learning schemes that accurately estimate the re-scaling factor are not effectively used in digital watermarking. We successfully use this neural network in the digital image watermarking field.
(3) With the fact that most of the papers in the image watermarking field test their methods only on several images, we use a large number of images to ensure the universality of our method. We test the ability to detect scaling factors on the datasets of RAISE, Boss, and Dresden. What is more, we randomly select 100 images from each of these photo databases to test the scheme performance on large color images which correspond to real-world situations. We also test our watermark scheme on high resolution color images that are consistent with real-world scenes.
(4) We embed an extra head watermark in another channel to find out the cutting offset. During the cutting position detection process, in order to speed up the searching process, we use four parallel processes starting in different positions.

The rest of this paper is organized as follows. In Section 2, some relevant preliminary terms are presented, including the scaling factor detection network, the datasets used, the quantization method, and the cutting position searching scheme. In Section 3, the detailed scheme, including the embedding and extracting processes, are described. Section 4 is the experimental part with analysis. Finally, the conclusions will be shown in Section 5.

## 2. Preliminaries

As we know, one major drawback of classical watermarking schemes is the lack of robustness to geometrical distortion. Thus, the detection of the watermark often requires a synchronization step to locate the embedded watermark in the content. In practical applications, if there is a watermarked image on the website, it may be downloaded by someone. He or she may resample this image and cut out some useful parts from it. Scaling and cropping the image at any position can disrupt the synchronization of the watermark, so how to resist scaling and cropping is the main task of robust watermarking algorithms. Although rotation is also possible, scaling and cutting are more common. Thus, in this paper, we focus on the scaling operations and the cutting operations. In our scheme, we consider using deep learning methods to obtain the scaling factor and use the head watermark searching method to obtain the cutting position. If we can obtain the scaling factor by neural networks, then we can easily restore the suspect watermarked image to its original size, and then we can search for the head watermark to locate the embedding position since the image may suffer from the cropping operation from any position. In this section, we introduce the related techniques of our scheme.

### 2.1. Scaling Factor Detection Network

Deep learning approaches, such as convolutional neural networks, have been a hot and useful method, have developed rapidly in recent years, and can automatically learn the parameters and extract the hidden features in many kinds of tasks, including scaling factor detection. The key layer of a convolutional neural network is the convolutional layer, which can be written as follows:

(1) $$h_{j}^{\left(n\right)}=\sum_{k=1}^{K}h_{k}^{\left(n-1\right)}*w_{kj}^{\left(n\right)}+b_{j}^{\left(n\right)}$$

where ∗ denotes the 2d convolution, $h_{j}^{\left(n\right)}$ is the j-th feature map of the n-th hidden layer and also to $h_{k}^{\left(n-1\right)}$, $w_{kj}^{\left(n\right)}$ is the k-th channel in the j-th filter in the n-th layer, and $b_{j}^{\left(n\right)}$ is the bias term of the j-th filter in the n-th layer. Different from the normal deep learning tasks, the scaling detection task recognizes the factor by focusing on the details in an image rather than the content of it. In fact, the content is a disturbance that should be suppressed before further learning. The constrained layer [23], however, is a distinguished solution to this problem, whose kernel has a sum of zero and a set of −1 in the center, as shown in Equation (2). Traditional methods in scaling detection always apply a filter on the image and then subtract the original one to obtain the details, and this process is mimicked by the constrained layer.

(2) $$\left\{\begin{array}{c} w_{kj}^{\left(n\right)}\left(0,0\right)=-1\\ \sum_{x,y\neq 0}w_{kj}^{\left(n\right)}\left(x,y\right)=1 \end{array}\right.$$

**Figure 1:**
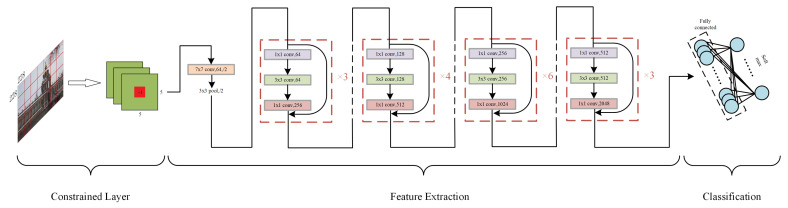
The proposed scaling factor detection model.

The structure of our scaling factor detection network is shown in Figure 1. We firstly put a constrained layer with a kernel of 5×5 in size and 3 in depth. Then we choose Resnet50 [28] as our backbone to extract deep features because it is a mature model and has been proved to perform well in many deep learning tasks. The Resnet50 network learns the residual but not the original parameters, which enable it to learn better and faster. The classification step includes a fully connected layer and a soft-max operation whose output is a scalar in the size of the class number, representing the possibilities of the input image belonging to different classes.

### 2.2. Datasets

In order to train a well-performing resampling detection network with high efficiency, we need to choose proper datasets. Obviously, the more images we use, the better performance of the neural network we can obtain, but the more time we need to consume, so we are supposed to choose a proper size for the dataset. In addition, in order to simulate the real-world environment, it is better to use images of high quality and large size. As a result, we combine Boss [29], RAISE [30], and Dresden [31] as our dataset and divide it into the training dataset and the validation dataset.

The training dataset consists of 1600 images from Boss, 1193 images from Dresden, and 800 images from RAISE and has 3593 images in total. And the validation dataset consists of 400 images from Boss, 298 images from Dresden, and 200 images from RAISE and has 898 images in total. They are shown in Table 1.

### 2.3. Quantization Method

**Figure 2:**
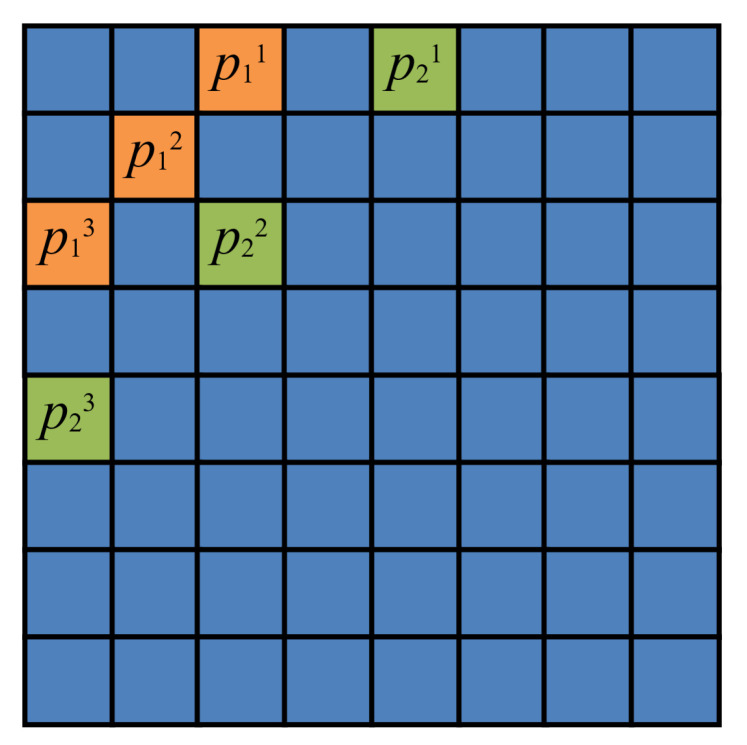
The quantization block.

We embed the watermark bits with the quantization method [32]. In every 8×8 block, we can embed 2 bits of information and for every bit, we modify three parameters to embed. As a result, we will change 6 parameter values for every 8×8 block. Assume that the parameters’ positions for bit 1 are $p_{1}^{1},p_{1}^{2},p_{1}^{3}$ and those for bit 2 are $p_{2}^{1},p_{2}^{2},p_{2}^{3}$, as shown in Figure 2.

We embed the information by quantizing the second-order difference, and the difference is calculated by Equation (3).

(3) $$\left\{\begin{array}{cc} d_{1} & =p_{1}^{1}+p_{1}^{3}-2\times p_{1}^{2}\\ d_{2} & =p_{2}^{1}+p_{2}^{3}-2\times p_{2}^{2} \end{array}\right.$$

The purpose of this quantization process is to put the difference into the middle of the nearest block with an odd index if the watermark bit is 0 and into the middle of the nearest block with an even index if the watermark bit is 1. Firstly, we calculate the Δd, which is related to the offset value of the parameters, and it is controlled by a parameter δ as well as the watermark bit. If the watermark bit is 0, Δd can be defined by Equation (4). If the watermark bit is 1, Δd can be defined by Equation (5).

(4) $$\Delta d=\left\{\begin{array}{cc} k\times \delta +\delta /2-d, & \mathrm{if}\;k\;\mathrm{is}\;\mathrm{even}\\ k\times \delta -\delta /2-d, & \mathrm{if}\;k\;\mathrm{is}\;\mathrm{odd}\;\mathrm{and}\;r<\delta /2\\ (k+1)\times \delta +\delta /2-d, & \mathrm{if}\;k\;\mathrm{is}\;\mathrm{odd}\;\mathrm{and}\;r\geq \delta /2 \end{array}\right.$$

(5) $$\Delta d=\left\{\begin{array}{cc} k\times \delta +\delta /2-d, & \mathrm{if}\;k\;\mathrm{is}\;\mathrm{odd}\\ k\times \delta -\delta /2-d, & \mathrm{if}\;k\;\mathrm{is}\;\mathrm{even}\;\mathrm{and}\;r<\delta /2\\ (k+1)\times \delta +\delta /2-d, & \mathrm{if}\;k\;\mathrm{is}\;\mathrm{even}\;\mathrm{and}\;r\geq \delta /2 \end{array}\right.$$

where *k* and *r* are defined by Equation (6), while ⌊·⌋ means the floor function.

(6) $$\left\{\begin{array}{cc} k & =\lfloor d/\delta \rfloor \\ r & =d-k\times \delta \end{array}\right.$$

After obtaining Δd, we can embed the watermark bit by changing the values of the block positions, which can be seen in Equation (7).

(7) $$\left\{\begin{array}{cc} p_{i}^{1^{\prime }} & =p_{i}^{1}+\Delta d/4\\ p_{i}^{2^{\prime }} & =p_{i}^{2}-\Delta d/4\\ p_{i}^{3^{\prime }} & =p_{i}^{3}+\Delta d/4 \end{array}\right.$$

### 2.4. Cutting Position Detection

A color image consists of three channels, and every channel has the same size. In the meanwhile, the attacks on images, except quantizing the color, will not mix the data of different channels. As a result, we can embed an extra head watermark in another channel to find out the cutting offset.

We use the DWT-DCT-based method to embed the head watermark. For the balance of efficiency and accuracy, firstly we apply the one-order/two-dimentional DWT to the image, and then we divide the image into blocks and embed the head watermark into the DCT coefficients of the block using the quantization method, as introduced in Section 2.3.

The main idea of the detection method is ergodic searching to find the position with maximal MSE, which is exactly the cutting offset. The MSE calculation formula is described in Equation (8).

(8) $$MSE=\frac{1}{mn}\sum_{i=0}^{m-1}\sum_{j=0}^{n-1}{\left[I\left(i,j\right)-K\left(i,j\right)\right]}^{2}$$

The details of searching process are as follows: firstly, we create a searching template by repeatedly splicing the known head watermark template until the size is bigger than the image size. Then we extract the watermarks from the image. We next move the start position from left to right, top to bottom, and cut the template into the same size with the image and then calculate the MSE between them. Finally, we find out the position with the maximal MSE, and that position will be the one we want to find.

**Figure 3:**
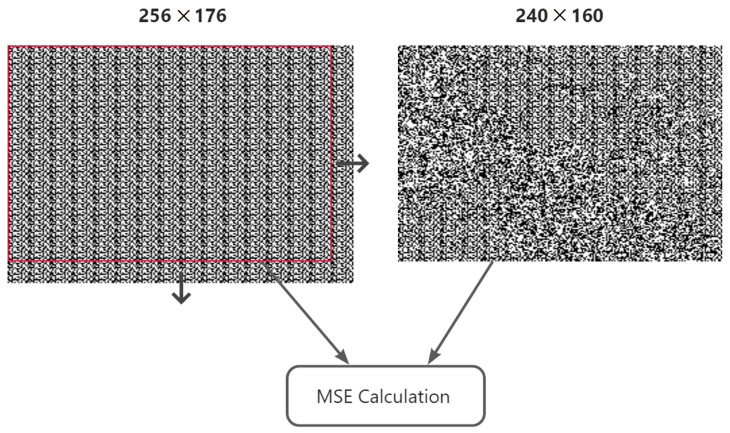
Finding cutting positions, where the arrows mean the window moving direction.

The block size of our method to entirely embed one watermark sequence is 256×256. Because of the one-order DWT and the 8×8 DCT block size, the maximum searching range is 16×16. We perform the searching and calculation as shown in Figure 3.

**Figure 4:**
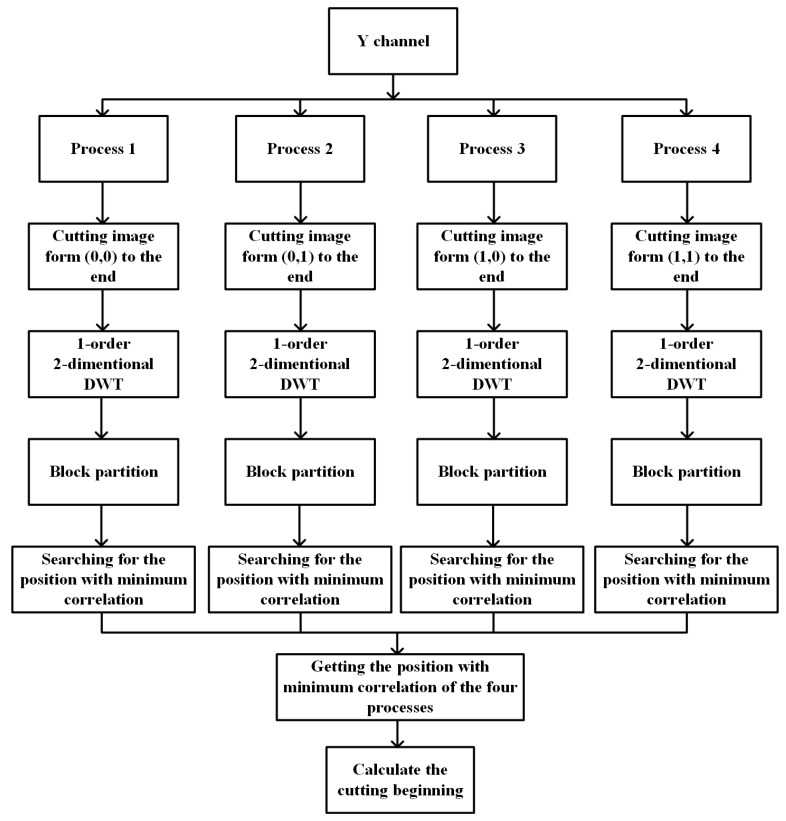
The whole searching process.

In order to speed up the searching process, we also use four parallel processes starting in different positions. As a result, this parallel searching will only cost one-fourth the time compared to the original one. The whole searching process is shown in Figure 4.

## 3. Proposed Scheme

**Figure 5:**
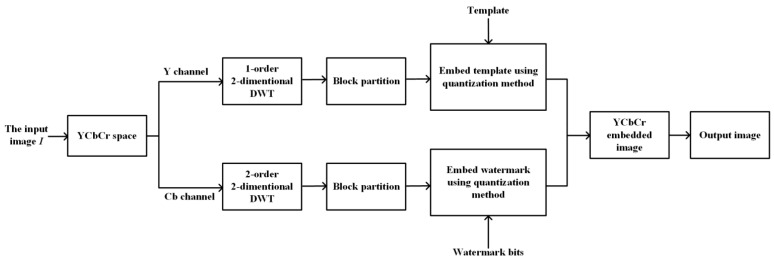
The process of embedding.

**Figure 6:**
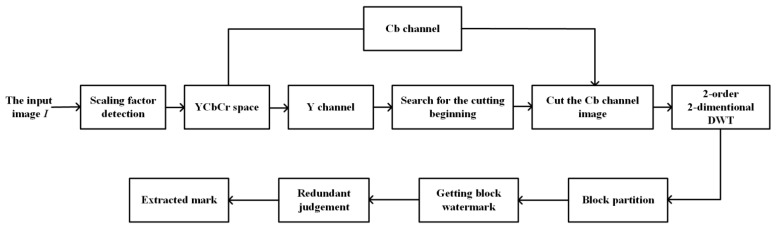
The process of extracting.

Robustness, imperceptibility, capacity, and computation complexity are the main factors we should consider when we design a watermarking scheme. In order to obtain a better robustness, we transform the image into the YCbCr color space and apply DWT to obtain the more stable low frequency data. What is more, we divide the image into blocks and repeatedly embed the same input watermark. As to the imperceptibility, we carefully select the parameters in the scheme, including the DCT positions to be embedded and the quantization value δ. We also choose a proper size of blocks to make this scheme have enough capacity. In addition, multiple processes are also applied to the extracting scheme in order to reduce the computation complexity. Figure 5 and Figure 6, respectively, display the detailed embedding and extracting schemes, and the specific processes are described in Section 3.1 and Section 3.2, respectively.

### 3.1. Watermarking Embedding Process

The watermark embedding process mainly includes two parts: information embedding and template embedding, which are performed in the Cb and Y channels, respectively. The information embedding process includes a two-order/two-dimensional DWT, block partition, block DCT, and coefficient quantization. The template watermark is a sequence with 256 bits. And the template watermark embedding process is almost the same as the information embedding process, except we replace the two-order DWT with a one-order DWT for the reason that the lower level DWT can reduce the number of head searching points so we can extract the watermark faster. The detailed embedding process is as follows:

Step 1: Color space transform. Firstly, we transform the original image I from the RGB color space into the YCbCr color space. Due to the fact that the human eye is more sensitive to brightness compared to chromaticity, YCbCr is more in line with human visual characteristics. In the watermarking algorithm, modifying the Y channel and Cb or Cr channel is relatively independent.

Step 2: Template embedding.

Step 2.1: Take the Y component $I_{Y}$ of the input image I; perform the one-order/two-dimensional DWT on $I_{Y}$ to obtain four subbands ${\mathrm{LL}}_{Y}$, ${\mathrm{LH}}_{Y}$, ${\mathrm{HL}}_{Y}$ and ${\mathrm{HH}}_{Y}$.

Step 2.2: Divide the lower frequency subband ${\mathrm{LL}}_{Y}$ into blocks ${\mathrm{LL}}_{i}$ of size 8×8, i=1,2,…,L, where *L* is the number of blocks. For each block ${\mathrm{LL}}_{i}$, we perform the two-dimensional DCT to obtain *L* DCT blocks ${\mathrm{DCTLL}}_{i}$ of size 8×8,i=1,2,…,L.

Step 2.3: For each 8×8 DCT block ${\mathrm{DCTLL}}_{i}$, embed two watermark bits using the quantization method as mentioned in Section 2. As shown in Figure 2, we change 6 parameter values for every DCT block. If the watermark bit is 0, Δd is defined by Equation (4). If the watermark bit is 1, Δd is defined by Equation (5). Then, we embed the watermark bit by changing the values of the block positions, as shown in Equation (7).

Step 2.4: The inverse DCT transform is applied to all DCT blocks to reconstruct new LL blocks, and then they are combined together to obtain the new LL subband, and finally, based on the new LL subband, together with the original LH, HL, and HH subbands, the inverse DWT transform is applied to obtain the new watermarked Y component $I_{\mathrm{YW}}$.

Step 3: Information watermark embedding.

Step 3.1: Take the Cb component $I_{\mathrm{Cb}}$ of the input image I; perform the two-order/two-dimensional DWT on $I_{Y}$ to obtain seven subbands ${\mathrm{LL}2}_{\mathrm{Cb}}$, ${\mathrm{LH}2}_{\mathrm{Cb}}$, ${\mathrm{HL}2}_{\mathrm{Cb}}$, ${\mathrm{HH}2}_{\mathrm{Cb}}$, ${\mathrm{LH}1}_{\mathrm{Cb}}$, ${\mathrm{HL}1}_{\mathrm{Cb}}$ and ${\mathrm{HH}1}_{\mathrm{Cb}}.$

Step 3.2: Divide the lower frequency subband ${\mathrm{LL}2}_{\mathrm{Cb}}$ into blocks $\mathrm{LL}2_{i}$ of size 8×8, i=1,2,…,M, where *M* is the number of blocks. For each block $\mathrm{LL}2_{i}$, we perform the two-dimensional DCT to obtain *M* DCT blocks $\mathrm{DCTLL}2_{i}$ of size 8×8,i=1,2,…,M.

Step 3.3: For each 8×8 DCT block $\mathrm{DCTLL}2_{i}$, embed two head watermark bits using the quantization method as mentioned in Section 2. As shown in Figure 2, we change six parameter values for every DCT block. If the watermark bit is 0, Δd is defined by Equation (4). If the watermark bit is 1, Δd is defined by Equation (5). Then, we embed the watermark bit by changing the values of the block positions, as shown in Equation (7).

Step 3.4: The inverse DCT transform is applied to all DCT blocks to reconstruct new LL2 blocks, and then they are combined together to obtain the new LL2 subband, and finally, based on the new LL2 subband, together with the original LH1, HL1, HH1, LH2, HL2, and HH2 subbands, the inverse DWT transform is applied to obtain the new watermarked Cb component $I_{\mathrm{CbW}}$.

Step 4: Channel merging. Finally, we merge the new watermarked Y channel $I_{\mathrm{YW}}$, the new watermarked Cb channel $I_{\mathrm{CbW}}$, and the unchanged Cr channel $I_{\mathrm{Cr}}$ into a new image, which is the final watermarked image $I_{W}$.

### 3.2. Watermarking Extraction Process

For the image to be extracted, firstly, we need to detect the scaling factor using the pre-trained network described in Section 2. After re-scaling the image into the original size, we transform the image into the YCbCr color space and use the Y channel to locate the cutting position. Considering the computation complexity, we use four parallel processes to search for the position with maximal MSE as the cutting position. Then, we cut the image and extract the Cb channel to extract the watermark. The detailed extracting process is described as follows:

Step 1: Scaling factor detection.

Step 1.1: We divide the suspect image $I_{\mathrm{sus}}$ to be detected into blocks and input them into the pre-trained scaling factor detection network. For each block, the network will output a scaling factor.

Step 1.2: The final scaling factor α is the one appearing most times over all blocks.

Step 1.3: Then, we rescale the image $I_{\mathrm{sus}}$ by the reciprocal of α and convert it into the image $I_{\mathrm{sus}}^{\prime }$ with the original size.

Step 2: Cutting position detection.

Step 2.1: Take the Y component $I_{Y}^{\prime }$ of the image $I_{\mathrm{sus}}^{\prime }$; by ergodically searching $I_{Y}^{\prime }$ to find the position with maximal MSE, we obtain the cutting position. The detailed method is given in Section 2.

Step 2.2: Cut the image $I_{\mathrm{sus}}^{\prime }$ based on this starting position to obtain the final image $I_{\mathrm{cut}}^{\prime }$ to be extracted.

Step 3: Watermark bits extraction.

Step 3.1: Take the Cb component $I_{\mathrm{Cb}}^{\prime }$ of the input image $I_{\mathrm{cut}}^{\prime }$; the watermark information is extracted by the cut Cb component. We firstly perform the two-order/two-dimensional DWT on it to obtain seven subbands, i.e., LL2, HL2, LH2, HH2, HL1, LH1, and HH1, and then divide the low-frequency subband ${\mathrm{LL}2}_{\mathrm{Cb}}^{\prime }$ into 8×8 blocks $\mathrm{LL}2_{i}^{\prime }$, i=1,2,…,M, where *M* is the number of blocks.

Step 3.2: DCT is performed on each block $\mathrm{LL}2_{i}^{\prime }$ to obtain the corresponding DCT block $\mathrm{DCTLL}2_{i}^{\prime }$. Then, we calculate the difference between DCT coefficients and figure out its belonging quantized interval with Equations (3) and (6).

Step 3.3: If the resulting *k* is even, the extracted watermark bit is 1; otherwise, if the resulting *k* is odd, the extracted watermark bit is 0.

Step 4: Redundant judgment.

Step 4.1: After extracting the watermark from every block, we need to decide the final watermark. For every bit of the final mark, if the number of 1s is larger than the number of 0s, then the final bit is set to 1; otherwise, it will be set to 0.

Step 4.2: When the redundant judgment is completed, the final watermark can be extracted.

## 4. Experimental Results

Our experiments were realized on an Intel(R) Core(TM) i5-7400 CPU, from Intel Corporation in Santa Clara, California, United States, with a Python framework. The scaling factor detection network is trained on a machine equipped with NVIDIA GeForce GTX 1080 Ti, from NVIDIA Corporation in Santa Clara, California, United States. The quantization factor δ is set to be 48.

**Figure 7:**
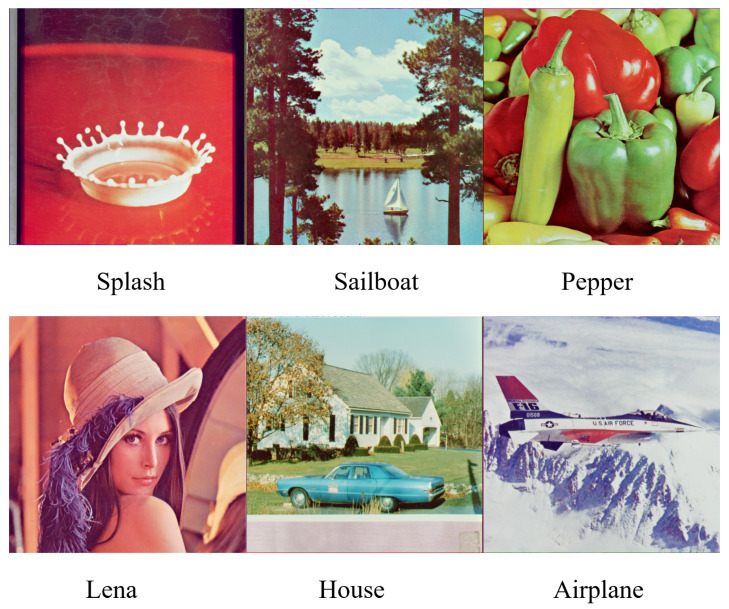
Host images.

The color images of size 512×512 in Figure 7 are selected as the host images to compare the performance of our watermarking scheme with others. To show the superiority of our scaling factor detection network, we test the ability to detect scaling factors on the datasets of RAISE [30], Boss [29], and Dresden [31]. What is more, we randomly select 100 images each from these photo databases to test the scheme performance on large color images which correspond to real-world situations. In order to show the superiority of the proposed scheme, we also compare with nine existing schemes, including seven transform-domain-based methods and two deep-learning-based methods. The seven transform-domain-based methods are as follows: (1) Ernawan and Ariatmanto’s DWT-DCT-based method [33]; (2) Wang et al.’s discrete Hartley-transform-based scheme [16], which mainly uses the image geometric features such as sides and angles to correct the attacked image; (3) Wang et al.’s robust periodic blind watermarking scheme [17] based on sub-block mapping and block encryption to enhance robustness under combined attack; (4) Kamili et al.’s two-channel method [6], which embeds robust and fragile watermarks into Y and Cb channels, respectively; (5) Wang et al.’s [34] PDTDFB magnitude and relative-phase-modeling-based method; (6) Yang et al.’s undecimated discrete wavelet-transform-domain-based method [35]; and (7) Wang et al.’s polar-harmonic-transform-based method [36]. The two deep-learning-based methods are: (1) Kumari et al.’s hybrid watermarking scheme based on DWT and SVD in addition to a deep belief neural network [25] and (2) Singh and Singh’s deep-learning-based watermarking algorithm [26].

### 4.1. Imperceptibility

The imperceptibility means people cannot distinguish the watermarked images from the original ones, which can be measured by the peak signal-to-noise ratio (*PSNR*), and structural similarity index metric (*SSIM*). The *PSNR* measures the similarity of the original image and the watermarked one, while the *SSIM* measures the structural similarity index between them. As for a color image, the *PSNR* can be defined in Equation (9). If the *PSNR* is larger than 30 dB, we assume that the imperceptibility is good.

(9) $$\begin{array}{cc} \; & PSNR=10log_{10}\left(\frac{MAX_{I}^{2}}{MSE}\right)\\ \; & MSE=\frac{1}{mn}\sum_{i=0}^{m-1}\sum_{j=0}^{n-1}{\left[I\left(i,j\right)-K\left(i,j\right)\right]}^{2} \end{array}$$

where *I* and *K* are the original and watermarked images, respectively, and $MAX_{I}$ is the possible maximal value of the image, which is 255 for a uint8 image.

The *SSIM* can be defined as in Equation (10). The bigger the *SSIM* is, the higher the imperceptibility the method has. And the maximal value is 1, which means there is no difference between two images.

(10) $$SSIM\left(x,y\right)=\frac{\left(2\mu_{x}\mu_{y}+c_{1}\right)\left(2\sigma_{xy}+c_{2}\right)}{\left(\mu_{x}^{2}+\mu_{y}^{2}+c_{1}\right)\left(\mu_{x}^{2}+\mu_{y}^{2}+c_{2}\right)}$$

where $\mu_{x}$ and $\mu_{y}$ means the average of *x* and *y*, $\sigma_{x}^{2}$ and $\sigma_{y}^{2}$ means the variance of *x* and *y*, and $\sigma_{xy}$ is the covariance between them.

The imperceptibility results of our scheme for different images are shown in Table 2. As we can see, the *PSNR* and *SSIM* obtained for the various host images are greater than 44.9 dB and 0.989, respectively, which means the proposed method is proficient in providing the watermarked images with high quality. The comparison of the average imperceptibility over six test images among different image watermarking methods can be seen in Table 3. From these results, we can see that our scheme has better imperceptibility than most of the existing schemes.

The reason why our algorithm can obtain better imperceptibility in PSNR is that our scheme is based on the DWT-DCT domain, and we only modify the DCT coefficients of the LL subband of DWT, and we carefully select the parameters in the scheme, including the DCT positions to be embedded and the quantization value δ. Ernawan and Ariatmanto’s method [33] obtained the best imperceptibility because it is also DWT-DCT-based. Their method is better than our method since we embed two watermarks (i.e., the template watermark and the information watermark) in both the Y and Cb channels, which brings much more distortion.

### 4.2. Robustness

Robustness is one of the most significant criteria in evaluating watermarking methods, which measures the ability to extract the watermark from the images under attack. We use the Lena image as the host image and compared the robustness testing results with other methods against general and geometric attacks. The robustness is evaluated by bit error rate (BER), which is defined as the ratio between the number of incorrect bits and the length of the watermark.

#### 4.2.1. Robustness against General Attacks

The general attacks we evaluated included adding salt and pepper noise, Poisson, average filtering, Gaussian filtering, median filtering, and JPEG compression with quality factors ranging from 60 to 90. The methods we choose to compare include [6,16,17,25,26,33,34,35,36]. Among these algorithms, three methods are most related to our schemes: Kamili et al. [6] proposed a two-channel method, embedding robust and fragile watermarks into the Y and Cb channels, respectively. And our scheme also embedded watermark information and search templates into these channels. Wang et al. [34] used PDTDFB magnitude and relative phase modeling to resist geometric attacks, which has the same purpose as our method. Ernawan et al. [33] embedded the watermark by modifying selected DWT-DCT coefficients, which has similarity with our watermark-embedding method. As a result, we choose those methods for the comparison. The comparison results can be seen in Table 4, showing that our method can resist all of these general attacks and performs better in most of the attacks. Our method has the best performance on the robustness to Poisson, average filtering, Gaussian filtering, and median filtering. However, our method is not very good at withstanding salt/pepper noise and JPEG compression.

The reason why our scheme can obtain the best robustness to filtering operations (average filtering, Gaussian filtering and median filtering) among the existing methods is that our method is based on DWT-DCT, where the DCT coefficients of the LL subband of DWT are used to embed the watermarks, while DWT-DCT coefficients are stable under the filtering attack, and thus the filtering operations have few effects on the embedded watermarks in the watermarked image. In fact, Ernawan and Ariatmanto’s method [33] also obtained the second-best robustness to filtering operations because it is also DWT-DCT-based. On the other hand, DWT-DCT coefficients are also stable under the adding of non-bipolar impulse noise attacks, and thus our scheme also has the best robustness to adding Poisson noise. However, our scheme does not have the best robustness to adding bipolar impulse noise, such as salt and pepper noise, since this kind of noise has great effects on DWT-DCT coefficients. In general, the DCT-based watermarking method is robust to JPEG compression, e.g., Ernawan and Ariatmanto’s method [33] has the best robustness to JPEG compression. In principle, our algorithm should also be very robust to JPEG compression. However, our scheme seems to be not very robust to JPEG compression, probably because, compared with [33], we perform two watermark embedding processes on both the Y and Cb components, and the information embedding process is performed in the Cb component, which is not more robust to JPEG compression than embedding only in the Y component.

Common image processing operations generally have effects on all pixels but reserve the content of the image, i.e., most of the common image processing operations have fewer effects on low-frequency components. Our scheme uses the DCT coefficients of the LL subband of DWT to embed watermarks, and when the common image processing operations are performed on the watermarked image, the DCT coefficients of the LL subband of DWT are modified by a small amount that is not enough to change the watermark information embedded, and thus the related watermark information can be extracted correctly. For salt and pepper noise, it changes some pixels to white pixels and changes some pixels to black pixels, and this modification may have great effects on the DCT coefficients of the LL subband of DWT, which may have effects on the extraction results.

#### 4.2.2. Robustness against Scaling Attacks

Scaling is a very common but harmful geometric attack. The methods we choose to compare include [6,16,17,25,26,33,34,35,36]. We compare the extracting results for Lena after scaling with factors of 90%, 120%, 140%, and 150%, as shown in Table 5. As the results show, our method has no error bits in this scenario, while other scaling-resistant schemes cannot extract an exactly correct watermark. Here, the authors who proposed the deep-learning-based method in [26] performed no experiments for scaling attacks, and maybe the scaling attacks were not considered during training. In [33], the authors only considered the rescaling attacks of 512-256-512 and 512-1024-512, since their method should know the scaling factor or restore the images to original sizes before watermark extraction. In our opinion, the scaling factor should be unknown or should be detected automatically before watermark extraction. In order to verify the universality of our scheme, we also perform experiments in all the 512×512 host images with a larger range of scaling factors. The results in Table 6 indicate that the proposed method has a good performance in resisting scaling attacks, and for most of the cases, we can extract no-error watermarks.

The reason why our scheme can obtain the best robustness to scaling is that our scheme uses a scaling detection neural network. This network is trained to focus on the detailed trace of the scaled images. Thus, our scheme can automatically and accurately obtain the scaling factor that the watermarked image may suffer. After rescaling the image by the reciprocal of the detected scaling factor, we can then accurately extract the watermark. Different from other algorithms that add template watermarks to resist scaling attacks, our scheme detects the scaling factor only based on the detection network that has been trained in advance. The second reason is that our DCT-DWT-based embedding operation can guarantee that the corresponding extraction operation has the ability to extract the watermark correctly from the rescaled watermarked image that is with the same size of the original image. As long as the scaling factor is correctly detected, after the rescaling operation, our extraction algorithm can correctly extract the watermark.

#### 4.2.3. Robustness against Cutting Attacks

Cutting or cropping is a kind of common attack in image processing. To evaluate the effectiveness of our scheme, the methods we choose to compare include [6,16,17,25,26,33,34,35,36]. We test the ability of our method to resist cutting attacks with factors 10%, 20%, 30%, and 50%, whose results are shown in Table 7. In the searching process, we will cut the extra part and only use the entity block to extract the watermark. Because the size of host images and blocks are 512×512 and 256×256, respectively, we will extract the watermark from the same bottom-right part whether the factor is 10% or 50%, resulting in the same BER in comparison. Here, the authors in [25,26] did not conduct experiments for cutting or cropping attacks since they did not use cutting or cropping attacks during training. The extraction results of the all six host images are shown in Table 8, and the average BER 0.26 proves the ability of our scheme to resist cutting attacks. From this table, we can see that for most test images, our method can 100% accurately extract the watermarks from cropped images. The reason is that our scheme uses a special searching scheme to find the cutting position for synchronization. It seems that the schemes in [17,33,36] have better results than our scheme. In fact, for most images, our scheme can 100% correctly extract the watermark after cutting, as shown in Table 8, while for many other schemes, they cannot 100% correctly extract the watermark for each image. The same value, 0.78, means that sometimes there is a fixed minimal step error during the search process. In future work, we will use better methods to search the cutting positions.

From Table 7, together with Table 8, we can see that our scheme has the relatively better and more stable robustness to cutting operations. The reason is that our scheme embeds a template watermark in the Y component and finds the position using maximal MSE searching in order to obtain the accurate cutting position. That is, we can find out the accurate cutting offset before watermark extraction. In addition, during the cutting position detection process, in order to speed up the searching process, we use four parallel processes starting in different positions.

### 4.3. Capacity and Security

For watermarking methods applied to copyright protection, robustness is the main performance consideration, while capacity is not the main performance consideration. To show the capacity performance, Table 9 lists the embedding capacity of ten methods, including [6,16,17,25,26,33,34,35,36].The capacity of the existing nine algorithms is either 32×32 or 64×64, while the capacity of our algorithm is divided into two parts: one part is for template watermarking (32×32×2=2048), and the other part is for information watermarking (16×16×2=512).

The security of watermark information is not of particular concern in this article. Our main concern is how to improve the robustness against scaling and cutting attacks. However, many techniques can be added to improve the security. For example, we can encrypt the information watermark before embedding. The coefficient positions can be also adopted as an embedding key. For all the nine methods compared, only Wang et al. [16] mentioned the security problem. In their paper, the advantages of the NP-hard problem in the RSA algorithm and large key space of an affine transform were exploited. The communication security was guaranteed, and the key information was protected from being stolen by attackers. A similar technique can be also adopted in our scheme.

### 4.4. Experiments for Large-Resolution Real-World Images

In the real world, we regularly use images with high resolution. As a result, experiments for high-resolution databases are of great importance and have practical significance. The databases we choose are Boss [29], RAISE [30], and Dresden [31], whose images are obtained by different cameras without after-processing.

#### 4.4.1. Robustness against Scaling Attacks

As to extracting the watermark from images attacked by scaling, firstly we need to detect the scaling factor using the pre-trained neural network described in Section 2. As a result, the factor detection accuracy is of great importance to the extraction accuracy. We test our resampling detection neural network using the three databases. The images are scaled by the factors ranging from 60% to 150% (where the factor 100% means there is no scaling operation on the images). The results are shown in Table 10, which reveals that our network is able to detect the scaling factor for most of the real-world images.

Then, we randomly select 100 images from each database, embed watermarks, scale the images, and extract the watermarks from them. The final BER results in Table 11 show the excellent performance in resisting scaling attacks.

#### 4.4.2. Robustness against Random Cutting Attacks

We test the ability of our method to resist cutting attacks in these large-resolution databases. We randomly choose 100 images from every database and calculate the BER of the watermark extracted from the images under random cutting with the factors ranging from 10% to 50%. The results can be seen in Table 12, which shows that our scheme has the ability to resist cutting attacks in most of the scenes.

#### 4.4.3. Robustness against Mixture Attacks

Our method is also able to extract the embedded watermark from the watermarked images attacked by scaling and cutting simultaneously. We adopt the randomly chosen 100 images from the Boss [29], RAISE [30], and Dresden [31] data sets, whose results are shown in Table 13, Table 14 and Table 15, respectively. The testing cutting factors begin with 0% and end with 50%, with a step of 10%. The testing scaling factors begin with 60% and end with 150%, with a step of 10%. As we can see, our method performs well in the mixture attack scenes.

## 5. Conclusions

In this paper, we proposed a robust image watermarking scheme based on the quantization of the DWT-DCT coefficients, which can resist not only normal attacks but also geometric attacks, like scaling and cutting. Our method resists the scaling attack by using a scaling detection neural network, which is trained to focus on the detailed trace of the scaled images. We found that this neural network can calculate the scaling coefficient and then perform the inverse transformation to restore the image with the original size. In order to obtain the cutting position, a template watermark is embedded in the Y component and the position is found by maximal MSE searching. We found that the proposed cutting positioning algorithm can effectively locate watermark information. According to the experimental results, the proposed scheme has excellent performance in the area of robust image watermarking and has practical significance in real-world scenes. In particular, we found that our algorithm can not only resist ordinary attacks but can also resist 50% cropping and 60–150% scaling mixed attacks. However, the main disadvantage of our method is that it is not robust to rotation attacks. Future research directions include: (1) combining other techniques to make our scheme robust to rotation attacks and hybrid geometric attacks; (2) designing a better deep neural network to deal with all kinds of geometric attacks, together with some common image processing operations.

## Figures and Tables

**Table 1:** The details of the training and testing datasets.

Training Set		Validation Set	
Boss	1600	Boss	400
RAISE	800	RAISE	200
Dresden	1193	Dresden	298
Total	3593	Total	898

**Table 2:** The imperceptibility measurement results of our scheme.

Images	PSNR (dB)	SSIM
Lena	44.9891	0.9932
Pepper	45.1291	0.9929
Airplane	45.0222	0.9917
Sailboat	44.9009	0.9946
Splash	45.0555	0.9893
House	44.9449	0.9938
Average	44.9852	0.9933

**Table 3:** Comparisons of imperceptibility among different methods.

Method	[6]	[16]	[17]	[25]	[26]
Average PSNR	41.213	41.003	40.318	41.702	44.480
Average SSIM	0.9929	0.9666	0.9918	0.9934	0.9997
Method	[33]	[34]	[35]	[36]	Our
Average PSNR	47.112	40.829	40.189	40.230	44.985
Average SSIM	0.9870	0.9910	0.9901	0.9917	0.9933

**Table 4:** The comparisons of robustness to general attacks among different methods (using Lena image).

Method	[6]	[16]	[17]	[25]	[26]
No Attack	0	0	0	0.54	0
Salt and Pepper (0.01)	11.06	0.06	2.35	7.36	1.25
Poisson	5.93	1.21	2.15	3.40	1.57
Average Filter 3×3	8.50	0.23	1.59	14.30	0.59
Gaussian Filter	5.74	0.10	0.25	10.13	0.45
Median Filter 3×3	7.89	0.04	1.25	15.13	0.23
JPEG (Q = 60)	0.02	0.32	0	4.56	5.48
JPEG (Q = 70)	0	0.21	0	1.23	4.79
JPEG (Q = 80)	0	0.14	0	0.45	2.43
JPEG (Q = 90)	0	0.11	0	0.12	1.24
Method	[33]	[34]	[35]	[36]	Our
No Attack	0	0.48	0	0	0
Salt and Pepper (0.01)	0.16	0.37	3.69	0.68	2.34
Poisson	6.84	1.35	2.40	1.72	0
Average Filter 3×3	0.29	1.78	7.40	3.15	0
Gaussian Filter	0	0.49	3.88	1.07	0
Median Filter 3×3	0.02	1.95	4.69	2.56	0
JPEG (Q = 60)	0	0.51	7.7	2.13	8.59
JPEG (Q = 70)	0	0.37	4.59	1.39	2.34
JPEG (Q = 80)	0	0.14	4.11	0.97	0
JPEG (Q = 90)	0	0.05	3.42	0.68	1.56

**Table 5:** The comparisons of robustness to scaling attacks among different methods (using Lena image).

Method	[6]	[16]	[17]	[25]	[26]
Scaling factor 50%	5.18	5.41	6.71	7.81	1.03 (1-0.5-1)
Scaling factor 90%	4.39	4.75	5.68	6.54	0.91 (1-0.9-1)
Scaling factor 120%	0.98	1.01	1.05	2.12	0.57 (1-1.2-1)
Scaling factor 140%	0.89	0.79	0.98	1.11	0.45 (1-1.4-1)
Scaling factor 150%	0.37	0.65	0.47	0.92	0.32 (1-1.5-1)
Method	[33]	[34]	[35]	[36]	Our
Scaling factor 50%	0.48 (1-0.5-1)	5.45	13.72	6.71	0
Scaling factor 90%	0.22 (1-0.9-1)	2.67	4.20	4.74	0
Scaling factor 120%	0.11 (1-1.2-1)	1.10	4.22	3.69	0
Scaling factor 140%	0.05 (1-1.4-1)	1.14	4.13	3.78	0
Scaling factor 150%	0.03 (1-1.5-1)	1.17	3.98	4.10	0

**Table 6:** The obtained BER values under scaling attacks with different factors for different host images.

Factor	60%	70%	80%	90%	100%	110%	120%	130%	140%	150%
Lena	0	0	0	0	0	0	0	0	0	0
Pepper	0.78	1.56	0	0	0	0	0	0	0	0
Airplane	0	1.56	0	0	0	0	0	0	0	0
Sailboat	0.78	0	0	0	0	0	0	0	0	0
Splash	0	0	0	0	0	0	0	0	0	0
House	0	0.78	0	0	0	0	0	0	0	0
Average	0.26	0.65	0	0	0	0	0	0	0	0

**Table 7:** The comparisons of robustness to cutting attacks among different methods (using Lena image).

Method	[6]	[16]	[17]	[25]	[26]
Cutting rate 10%	5.45	6.13	0	2.78	4.79
Cutting rate 20%	11.75	10.79	0	5.12	10.48
Cutting rate 30%	17.21	16.75	0.81	8.41	16.12
Cutting rate 50%	25.93	24.51	1.31	11.23	23.79
Method	[33]	[34]	[35]	[36]	Our
Cutting rate 10%	0.05	0.28	3.22	0.71	0.78
Cutting rate 20%	0.10	0.45	3.86	0.61	0.78
Cutting rate 30%	0.18	0.69	4.93	0.61	0.78
Cutting rate 50%	0.23	1.13	17.6	0.90	0.78

**Table 8:** The obtained BER values under cutting attacks with different factors for different host images.

Cutting Factor	10%	20%	30%	40%	50%
Lena	0.78	0.78	0.78	0.78	0.78
Pepper	0	0	0	0	0
Airplane	0	0	0	0	0
Sailboat	0.78	0.78	0.78	0.78	0.78
Splash	0	0	0	0	0
House	0	0	0	0	0
Average	0.26	0.26	0.26	0.26	0.26

**Table 9:** The comparisons of the embedding capacity among different methods (using Lena image of size 512×512).

Method	[6]	[16]	[17]	[25]	[26]
Capacity(bits)	4096	1024	1024	4096	1024
Method	[33]	[34]	[35]	[36]	Our
Capacity(bits)	1024	4096	4096	4096	2560

**Table 10:** The resampling factor detecting accuracy for databases.

Database	BOSS	RAISE	Dresden
60%	99.65%	85.90%	99.66%
70%	100%	99.30%	100%
80%	100%	100%	100%
90%	100%	100%	100%
100%	100%	100%	100%
110%	100%	100%	100%
120%	100%	100%	100%
130%	100%	100%	100%
140%	100%	100%	100%
150%	100%	100%	100%

**Table 11:** The BER for datasets under scaling attacks.

Database	BOSS	RAISE	Dresden
60%	0	4.8203	0.5859
70%	0	1.9688	0.4531
80%	0	0.5391	0
90%	0	0	0
100%	0	0	0
110%	0	0	0
120%	0	0	0
130%	0	0	0
140%	0	0	0
150%	0	0	0

**Table 12:** The BER for datasets under cutting attack.

Factor	No Cutting	10%	20%	30%	40%	50%
BOSS	0	0	0	0	0	0
RAISE	0	0	0.4844	0	0	0.5469
Dresden	0	0	0	0	0	0

**Table 13:** The BER for BOSS for mixture attacks of scaling and cutting.

	0%	10%	20%	30%	40%	50%
60%	0	0	0	0.1875	0.7930	0.5391
70%	0	0	0	0	0.4805	0.2539
80%	0	0	0	0	0	0.5156
90%	0	0	0	0	0	0.3203
100%	0	0	0	0	0	0
110%	0	0	0	0	0	0.2695
120%	0	0	0	0	0	0
130%	0	0	0	0	0.3281	0
140%	0	0	0	0	0	0.2109
150%	0	0	0	0	0	0

**Table 14:** The BER for RAISE for mixture attacks of scaling and cutting.

	0%	10%	20%	30%	40%	50%
60%	4.8203	6.9063	8.2578	5.9844	9.4922	8.5313
70%	1.9688	0.9766	1.0078	1.9688	3.0781	3.4766
80%	0.5391	0	0	2.3750	0	0.4844
90%	0	0	0	0	0	0
100%	0	0	0.4844	0	0	0.5469
110%	0	0	0	0.5313	0.5156	0
120%	0	0	0	0	0	0
130%	0	0	0	0	0.5313	0
140%	0	0	0	0	0	0
150%	0	0	0	0	0	0

**Table 15:** The BER for Dresden for mixture attacks of scaling and cutting.

	0%	10%	20%	30%	40%	50%
60%	0.5859	0.5078	0.4609	0.5313	1.7578	0.5703
70%	0.4531	0.4688	0.5625	0.4453	0.9531	1.0078
80%	0	0	0	0	0	0
90%	0	0	0	0	0	0.0016
100%	0	0	0	0	0	0
110%	0	0	0	0	0	0
120%	0	0	0	0	0	0.0008
130%	0	0	0	0	0	0.0008
140%	0	0	0	0	0	0
150%	0	0	0	0	0	0.0008

## Data Availability

Not applicable.
